# Inhibition of STAT3 in gastric cancer: role of pantoprazole as SHP-1 inducer

**DOI:** 10.1186/s13578-018-0248-9

**Published:** 2018-09-06

**Authors:** Jin Sung Koh, Moon Kyung Joo, Jong-Jae Park, Hyo Soon Yoo, Byung Il Choi, Beom Jae Lee, Hoon Jai Chun, Sang Woo Lee

**Affiliations:** 1 Division of Gastroenterology, Department of Internal Medicine, Korea University College of Medicine Guro Hospital, 148, Gurodong-ro, Guro-gu, Seoul, 152-703 Republic of Korea; 2 Division of Gastroenterology, Department of Internal Medicine, Korea University College of Medicine Anam Hospital, 73, Inchon-ro, Seongbuk-gu, Seoul, 136-705 Republic of Korea; 3 Division of Gastroenterology, Department of Internal Medicine, Korea University College of Medicine Ansan Hospital, 123, Jeokgeum-ro, Danwon-gu,, Ansan-si, Gyeonggi-do 425-707 Republic of Korea

**Keywords:** Pantoprazole, Gastric cancer, SH2-containing protein tyrosine phosphatase 1, Signal transducer and activator of transcription 3

## Abstract

**Background:**

We investigated the inhibitory effect of pantoprazole on signal transducer and activator of transcription 3 (STAT3) activity and invasiveness of gastric adenocarcinoma cells, and the role of SH2-containing protein tyrosine phosphatase 1 (SHP-1) in mediating role.

**Methods:**

We used AGS and MKN-28 cells because of reduced SHP-1 and preserved p-STAT3 expression. Western blot, wound closure assay, Matrigel invasion assay and 3-D culture invasion assay were performed. Pharmacologic inhibitor of SHP-1 and siRNA were used for validation of the role of SHP-1.

**Results:**

We observed that pantoprazole at 40, 80, and 160 μg/ml upregulated SHP-1 and downregulated p-STAT3 expression in a dose-dependent manner in AGS and MKN-28 cells. Furthermore, pantoprazole significantly downregulated mesenchymal markers (Snail1 and vimentin), upregulated epithelial marker (E-cadherin), and inhibited migration and invasion of AGS and MKN-28 cells. To validate the role of SHP-1 in inhibition of STAT3 activity by pantoprazole in gastric cancer cells, we performed pharmacologic inhibition (pervanadate) or knockdown of SHP-1 before pantoprazole treatment, which significantly attenuated the suppression of p-STAT3 and anti-migration and invasion effect by pantoprazole in AGS cells. In xenograft tumor model, tumor volume was significantly reduced by intraperitoneal injection of pantoprazole, with upregulation of SHP-1 and downregulation of p-STAT3, which were attenuated by concomitant injection of pervanadate.

**Conclusion:**

Our data suggest that the inhibitory effect of pantoprazole on cellular migration and invasion might be through inducing SHP-1 in gastric cancer cells.

## Background

Gastric cancer is one of the most frequently diagnosed cancers. It is the third leading cause of cancer-related death, and annually 720,000 gastric cancer-related deaths have been reported worldwide [1]. If gastric cancer is diagnosed in advanced stage, especially with distant metastasis, chance for complete remission is getting lower dramatically. The overall survival of non-curable stage IV gastric cancer remains under 18 months [2]. Thus, understanding the pathophysiologic mechanism about tumor invasion and progression is an essential step toward complete conquest of gastric cancer. It is known that Janus kinase 2 (JAK2)/signal transducer and activator of transcription 3 (STAT3) signaling pathway is induced in various biologic reactions related to gastric cancer, including inflammatory process caused by chronic *Helicobacter pylori* (*H. pylori*) infection and interaction between gastric epithelium and microenvironmental stromal cells to promote migration or invasion of cancer cells. Thus, inhibiting JAK2/STAT3 signaling seems to be a reasonable option for controlling multiple steps involved in gastric carcinogenesis and invasion [3]. Constitutive activation of STAT3 is closely related to poor prognosis of gastric cancer. A recent meta-analysis has clearly shown that elevated p-STAT3 activity is not only significantly associated with poor prognosis of stomach cancer patients, but with undifferentiated type and lymph node metastasis [4].

SH2-containing protein tyrosine phosphatase 1 (SHP-1), a non-receptor type protein tyrosine phosphatase, can dephosphorylate STAT3 and JAK2 which in turn will down regulate STAT3 activity to block various STAT3 mediated signaling pathways [5]. However, concerning the expression of SHP-1 in stomach tissue, data are very lacking. We have previously investigated promoter hypermethylation and gene expression of SHP-1 in most of gastric cancer cells and functional effects of SHP-1 on JAK2/STAT3 pathway [6]. Because protein or gene expression level of SHP-1 is very weak in most of stomach cancer tissues, SHP-1-induction strategy appears to be reasonable to effectively inhibit constitutive STAT3. We focused on pantoprazole, which is not only a well-known proton pump inhibitor (PPI), but has several unexpected effects such as anti-tumor effects. We investigated the anti-invasive effect of PPZ by modulating SHP-1/JAK2/STAT3 signaling axis in such effect.

## Methods

### Reagents and cell line

Pantoprazole (purity > 98%) was purchased from Sigma-Aldrich (St. Louis, MO, USA), and human stomach cancer cell lines (AGS, MKN-28) were obtained from Korean Cell Line Bank (Seoul National University, Seoul, Korea). Cells were cultured in RPMI (Gibco, Carlsbad, CA, USA) supplemented with 10% heat-inactivated FBS (Gibco) and penicillin/streptomycin (1.0%, Gibco-BRL). Cells were incubated at 37 °C in a humidified atmosphere with 5% CO_2_.

### Water-soluble tetrazolium salt-1 (WST-1) cell proliferation assay

Anti-proliferative effect of pantoprazole was measured by using a commercial WST-1 assay kit (EZ-CYTOX, Dogen, Seoul, Korea) according to the manufacturer’s instructions [7]. Briefly, 1 × 10^4^ of AGS cells per well were cultured in 96-well plates for 24 h followed by treatment with 40, 80, and 160 μg/ml pantoprazole for 24 and 48 h. After pantoprazole treatment, 10 μl of WST was added to each well for 4 h and the absorbance at 450 nm was measured. All experiments were performed in triplicates.

### Wound closure assay

After pharmacologic intervention or transfection for indicated times, cells were seeded onto a 6-well plate, and a monolayer wound was made on the bottom of the chamber with a 200-μl pipette tip. Vertical distance between both ends of the wound was measured at three random area.

### Matrigel invasion assay

Following pharmacologic treatment or transfection for indicated times, 4 × 10^4^ cells per well were seeded into 24-well Matrigel Invasion Chambers (BD Biosciences, Franklin Lakes, NJ, USA) with 2% FBS medium, while 10% FBS was added to lower wells. After 24 h, filter membrane was stained with crystal violet, and the positive invading cells were counted at five randomly selected areas under 20× magnification.

### 3-D culture spheroid cell invasion assay

The assay was performed by using a 96-well 3-D spheroid BME cell invasion assay kit (Trevigen, Gaithersburg, MD, USA). Briefly, after pharmacologic intervention or transfection, 2 × 10^3^ cells were suspended in extracellular matrix and added to on the 96-well spheroid formation plate. After 3 days, invasion matrix and medium containing invasion modulating compounds were added, and the shape of spheroid in each well were observed by using a 4 × phase-contrast microscope. The area of spheroids were measured and analyzed using open source software (ImageJ).

### Western blot analysis

Rabbit polyclonal IgG antibody against human SHP-1 (sc-287) and human β-actin (sc-47778) and a mouse monoclonal IgG antibody against Snail1 (sc-10433) were purchased from Santa Cruz Biotechnology, Inc. (Santa Cruz, CA, USA). Mouse monoclonal IgG antibodies against human STAT3 (#9139) and p-STAT3 (Tyr705, #4113), rabbit polyclonal antibodies against human JAK2 (#3230), p-JAK2 (Tyr1007/1008, #3771), E-cadherin (#3195), and a rabbit monoclonal antibody against human vimentin (#5741) were purchased from Cell Signaling Technology (Beverly, MA, USA). A total of 80–100 μg of cell lysate protein was extracted and loaded into SDS-PAGE gel. Primary antibodies were diluted at a ratio of 1:1000 in blocking buffer (Tris-buffered saline with Tween-20; Biosesang, Gyeonggi, Korea) containing 5% skim milk (Difco; Becton–Dickinson and Co., Sparks, MD, USA). Probed membranes were incubated at 4 °C overnight, and then incubated with goat anti-mouse or anti-rabbit IgG as a secondary antibody at room temperature for 1 h. Bands were visualized by exposing membranes to enhanced chemiluminescence (Perkin-Elmer, Waltham, MA, USA) for 1 min.

### Immunofluorescence

AGS cells or 4-μm sections of xenograft tumor tissue were plated on a slide glass, which was dried for 15 min at room temperature and fixed with 3.7% formaldehyde. After washing with water, slides were incubated with a rabbit monoclonal anti-human SHP-1 IgG antibody (Abcam, ab124942, Cambridge, UK), a rabbit monoclonal anti-human vimentin IgG antibody, a rabbit polyclonal anti-human E-cadherin IgG antibody, a mouse monoclonal anti-human Snail1 IgG antibody or a mouse monoclonal anti-human p-STAT3 IgG antibody (1/100 dilution) at 4 °C overnight. After washing, slides were incubated with goat anti-mouse IgG (green) or goat anti-rabbit IgG (green) (1/500 dilution) at room temperature for 1 h. Counterstaining was performed by incubating with 4′,6-diamidino-2-phenylindole (DAPI, blue) (Vectashield, Vector Laboratories, Burlingame, CA, USA) at room temperature for 5 min. Slides were observed under a confocal microscope (LSM 700, Carl Zeiss, Oberkochen, Germany) and images were captured by using a high-resolution digital camera (Carl Zeiss).

### Transfection

SHP-1 (sc-29478) and control (sc-37007) siRNA were purchased from Santa Cruz Biotechnology. For transient small interfering RNA (siRNA) transfection, cells with 70–80% of confluence were transfected by using 2 μg of plasmid using Lipofectamine 2000 (Invitrogen, Life Technologies). After 24 h of transfection, cells were collected and used for further functional analyses.

### Xenograft tumor in nude mice

Six-week-old male Balb/C athymic nude mice (nu/nu) were housed and maintained under pathogen free conditions. Xenograft tumors were produced following a previously described protocol [8]. At brief, 2 × 10^6^ AGS cells mixed with Matrigel were injected subcutaneously in both shoulders of each mouse. After 2 weeks of window period, mice were intraperitoneally injected with 180 mg/kg/week of pantoprazole with or without 18 mg/kg/week of pervanadate for 8 weeks. Mice in the control group were intraperitoneally injected with normal saline using the same injection schedule. Tumor size was measured two times per week by using a slide caliper. Tumor volume was calculated with the following formula; 0.44 × A × B^2^ (A = longer diameter, B = perpendicular diameter of A). This experiment was approved by the Ethic Committee of Korea University Laboratory Animal Research Center (Reference No. KOREA-2017-0189).

### Immunohistochemistry

Immunohistochemistry (IHC) staining of xenograft tumor was described in previous studies [9]. Briefly, samples were fixed in 10% formalin, embedded in paraffin, and sectioned in 4-μm in thickness. Sections were attached to slides, dried and deparaffinized overnight, and rehydrated. Slides were incubated with anti-SHP-1 or anti-p-STAT3 antibody at room temperature for 30 min followed by the addition of an avidin–biotin–peroxidase complex. IHC stains were observed under a motorized microscope and images were captured by using a digital camera.

### Statistical analysis

We used SPSS software ver. 19.0 (SPSS, Inc., Chicago, IL, USA) for statistical analyses. Continuous data were presented as median ± standard deviation. Student’s *t*-test was used for comparison of data, and a p-value < 0.05 was considered statistical significant.

## Results

### Pantoprazole upregulates SHP-1 expression and downregulates p-JAK2/p-STAT3 in gastric cancer cells

**Fig. 1:**
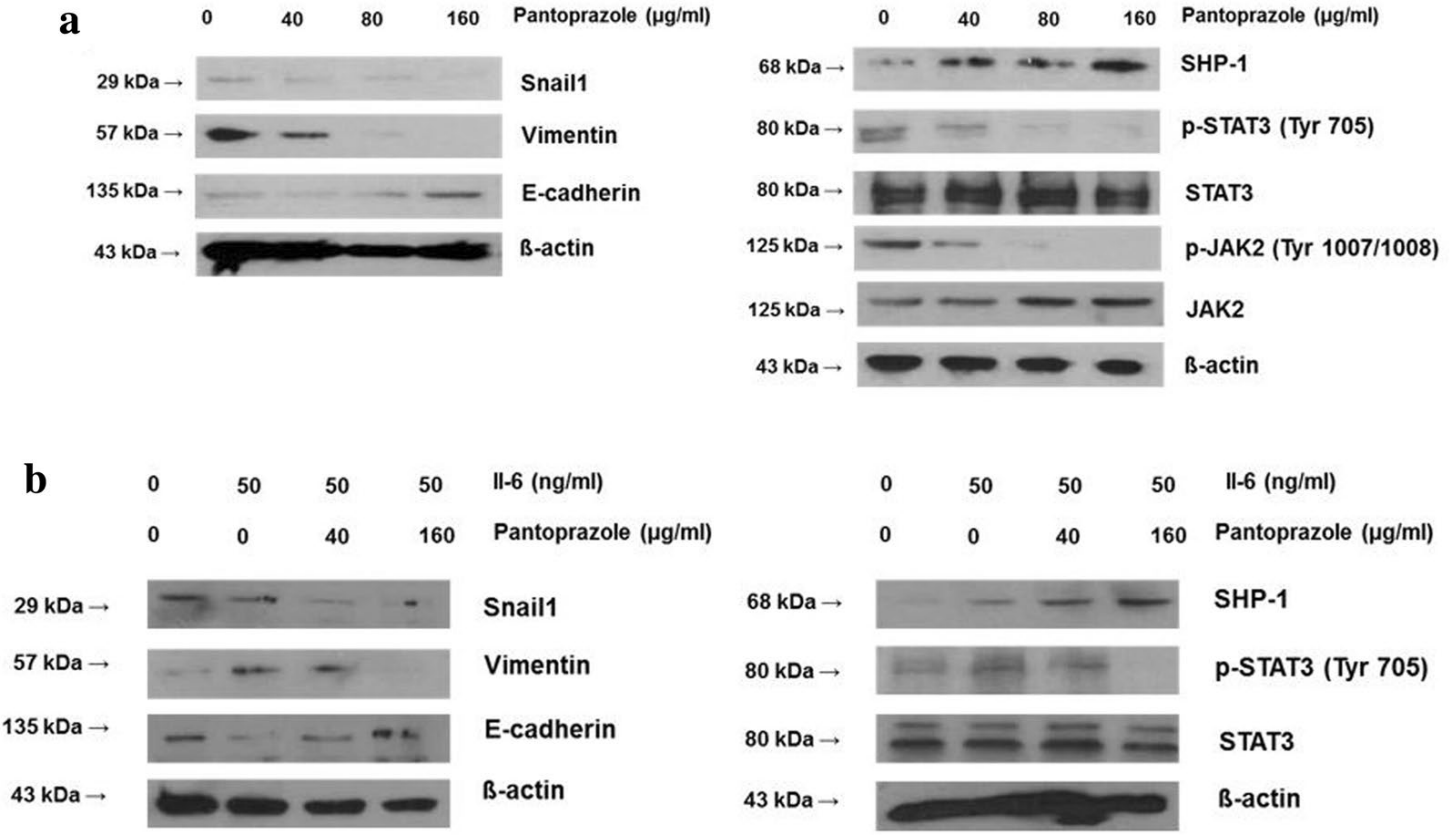
Pantoprazole upregulates SHP-1 and downregulates p-STAT3 in gastric cancer cells. **a** Western blotting for AGS cells after treatment with pantoprazole at 40, 80, and 160 μg/ml for 48 h. **b** Western blotting from MKN-28 cells after stimulation with 50 ng/ml of IL-6 for 1 h, followed by treatment with pantoprazole

**Fig. 2:**
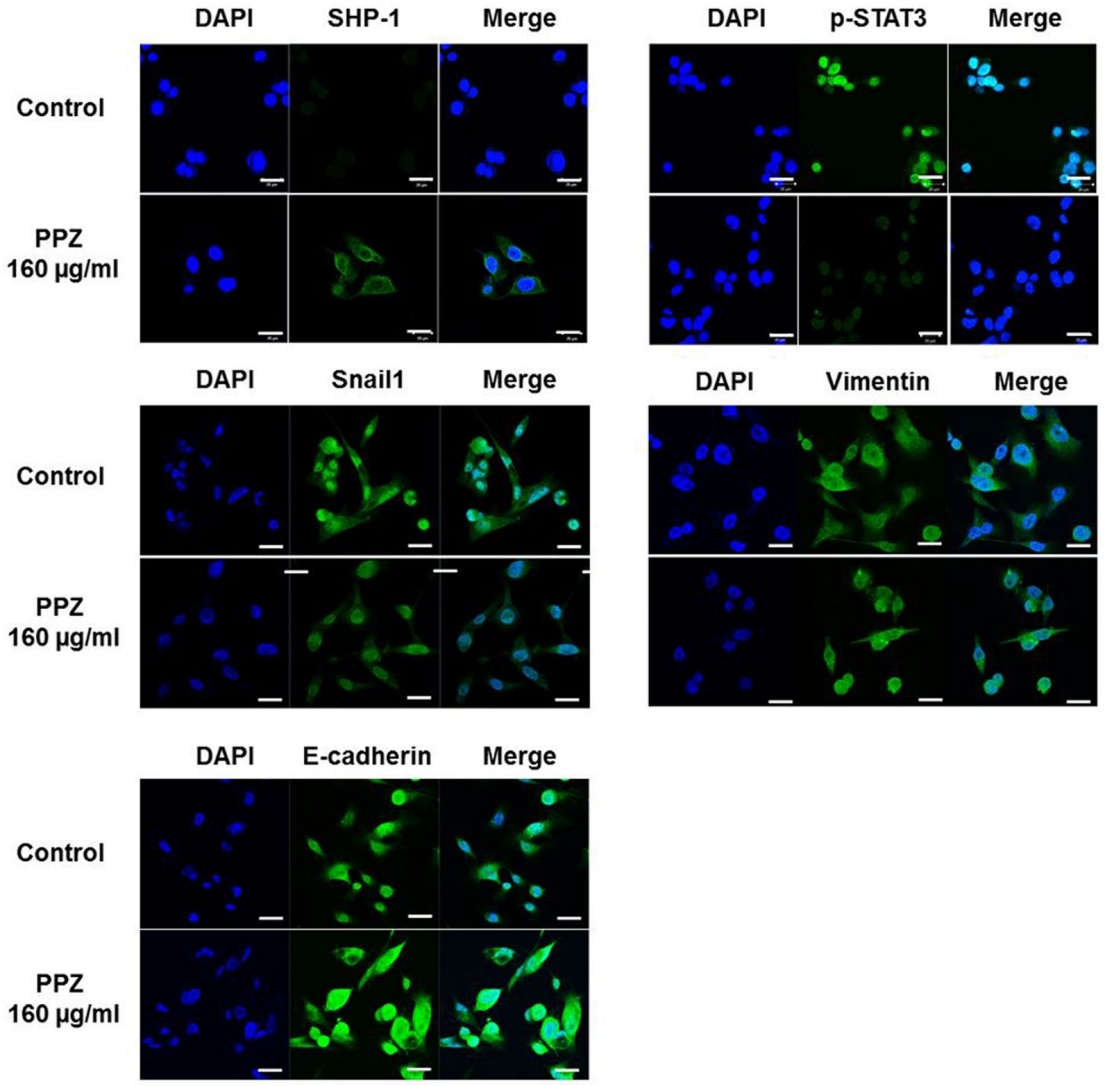
Immunofluorescence of SHP-1 and p-STAT3 after 48 h treatment with pantoprazole. AGS cells are stained with SHP-1 (green), p-STAT3 (green), Snail1 (green), vimentin (green), E-cadherin (green) and 4′,6-diamidino-2-phenylindole (DAPI) (blue). White bar indicates 20 μm; PPZ, pantoprazole

First, we observed the modulating effect of pantoprazole on SHP-1/p-JAK2/p-STAT3 axis in AGS cells and IL-6-stimulated MKN-28 cells. We firstly used AGS cells due to weak expression of SHP-1, constitutive p-JAK2/p-STAT3 expression and mesenchymal phenotype. We observed that 48 h treatment with pantoprazole at 40, 80, and 160 µg/ml upregulated SHP-1 expression and downregulated p-JAK2/p-STAT3 expression in AGS cells in a dose-dependent manner, which in turn downregulated Snail1 and vimentin (markers of mesenchymal transition) but upregulated E-cadherin, an epithelial marker in epithelial cells (Fig. 1a) [10]. We also investigated whether pantoprazole could inhibit IL-6-stimulated p-STAT3 activity in MKN28 cells. As expected, treatment with pantoprazole at 40, 80, and 160 µg/ml downregulated IL-6-stimulated p-STAT3 expression but upregulated SHP-1 expression. Mesenchymal markers (Snail1, vimentin) were downregulated while epithelial marker (E-cadherin) was upregulated by pantoprazole treatment (Fig. 1b). We performed immunofluorescence staining to visualize the change in expression of SHP-1, p-STAT3 and EMT markers by pantoprazole in AGS cells. Results showed upregulation of cytosolic staining of SHP-1 but attenuation of nuclear staining of p-STAT3 in AGS cells after pantoprazole treatment, Modulation of EMT markers (upregulation of E-cadherin and downregulation of vimentin, Snail1) were also observed (Fig. 2). Taken together, our data suggest that pantoprazole modulate expression of p-JAK2/p-STAT3 (downregulation) and SHP-1 (upregulation) and EMT markers in gastric cancer cells.

### Pantoprazole attenuates cellular proliferation, migration and invasion in gastric cancer cells

**Fig. 3:**
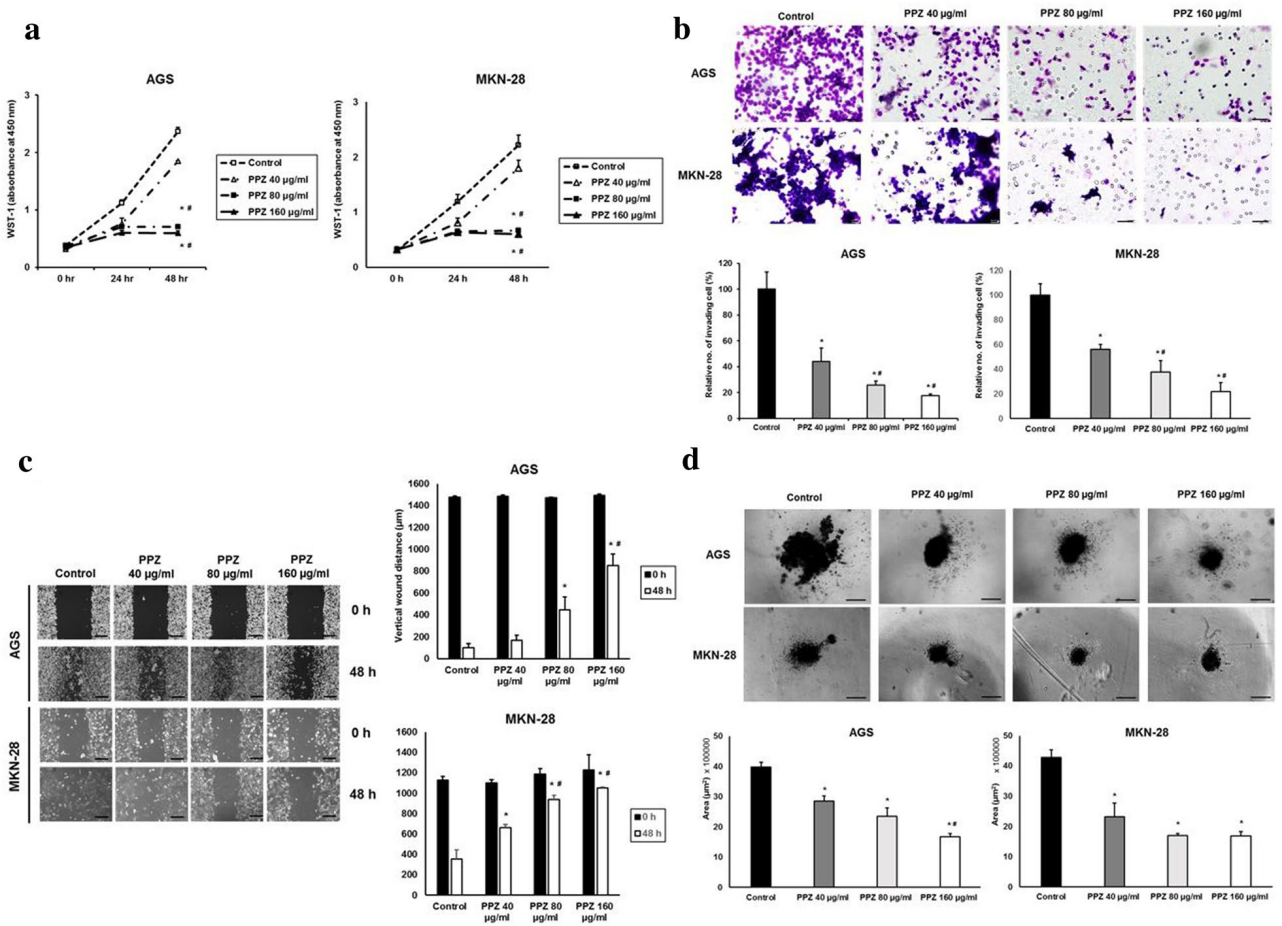
Pantoprazole attenuates proliferation, migration, and invasion of gastric cancer cells. **a** WST-1 cell proliferation assay. All experiments were performed in triplicates. *P < 0.05, compared with control; ^#^P < 0.05, compared with pantoprazole at 40 μg/ml (n = 3). **b** Matrigel invasion assay. Upper panel: representative images of invasion assay. Black bar indicates 50 μm. Lower panel: comparison of invading cells. Data are presented as mean ± standard deviation. Cell counting was performed in five randomly selected separate areas under ×20 magnification. *P < 0.05, compared with control; ^#^P < 0.05, compared with pantoprazole at 40 μg/ml (n = 5). **c** Wound closure assay. Left panel: representative images of assay. Black bar indicates 500 μm. Right panel: Comparison of vertical wound distance. Data are presented as mean ± standard deviation. All experiments were performed in triplicates. *P < 0.05, compared with control; ^#^P < 0.05, compared with pantoprazole at 40 μg/ml (n = 3). **d** 3-D culture spheroid cell invasion assay. Upper panel: representative images of 3-D culture spheroid invasion assay. Black bar indicates 200 μm. Lower panel: comparison of area of spheroids. Data are presented as mean ± standard deviation. All experiments were performed in triplicates. *P < 0.05, compared with control; ^#^P < 0.05, compared with pantoprazole at 40 μg/ml (n = 3). PPZ, pantoprazole

We investigated the inhibitory effect of pantoprazole on proliferation migration and invasion of AGS and MKN-28 cells. WST-1 assay showed that treatment with pantoprazole at 80 and 160 µg/ml for 48 h significantly inhibited cellular proliferation (Fig. 3a). Pantoprazole treatment at 40, 80, and 160 µg/ml also significantly decreased the number of invading cells (Fig. 3b) and increased vertical wound distance (Fig. 3c) in a dose-dependent manner. We then performed 3-sheroid cell invasion assay to visually confirm its anti-invasion effect. Results showed that treatment with pantoprazole at 40, 80, and 160 µg/ml dramatically decreased the spindle-like projection of cells into the surrounding matrix compared to control (Fig. 3d). Taken together, our data suggest that treatment with pantoprazole can attenuate invasion and migration of AGS and MKN-28 cells. Such effects are closely associated with its inhibition on STAT3 activity [11].

### Inhibition of SHP-1 attenuates the downregulation of STAT3 activity and inhibition of cellular migration and invasion caused by pantoprazole

**Fig. 4:**
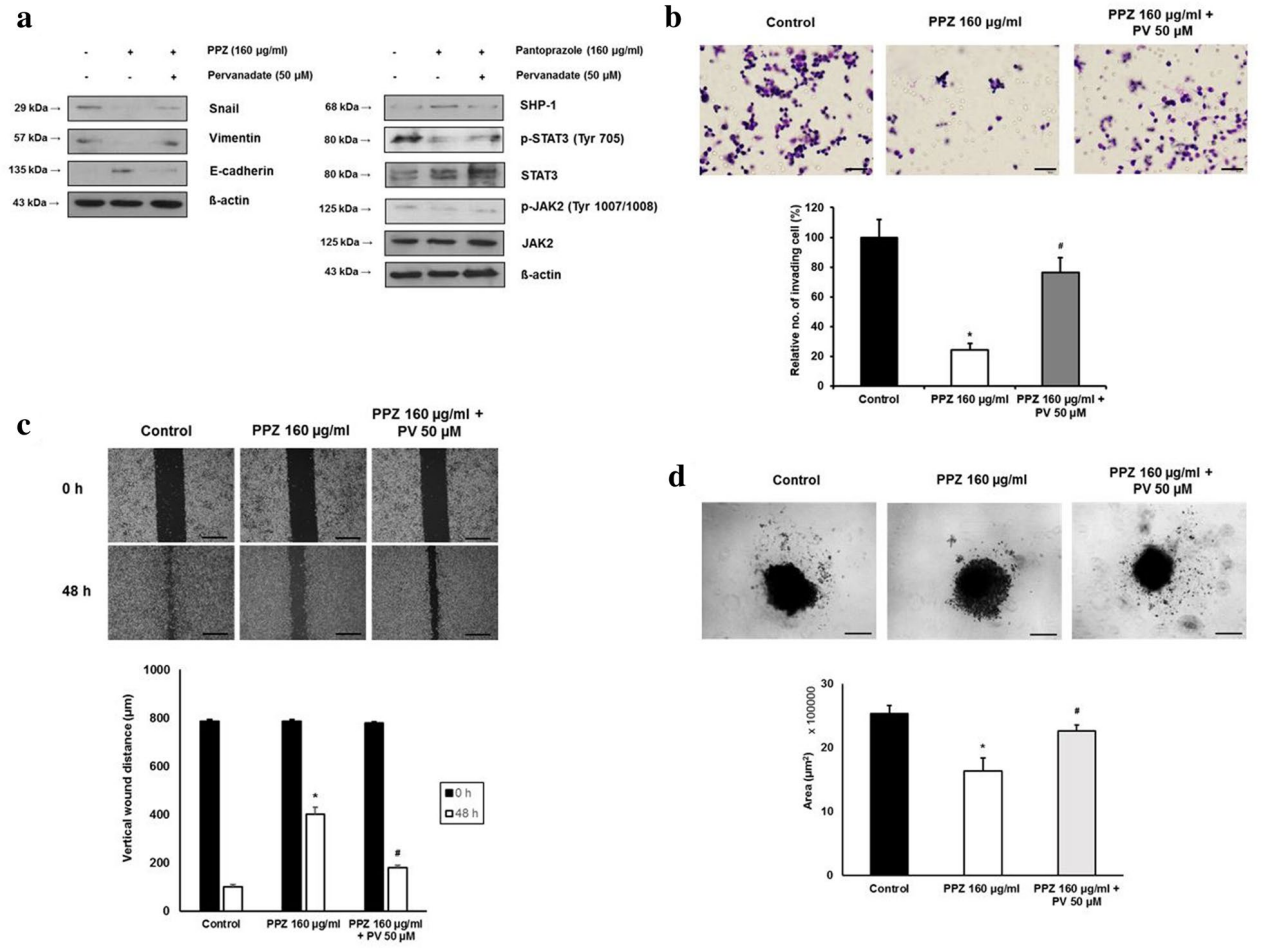
Inhibitory effects of pantoprazole on STAT3 activity, cellular migration, and invasion are attenuated by phosphatase inhibitor. **a** Western blot after 48 h of treatment with pantoprazole at 160 μg/ml with or without pretreatment with 50 nM pervanadate. **b** Matrigel invasion assay. Upper panel: representative images of assay. Black bar indicates 50 μm. Lower panel: Comparison of invading cells. Data are presented as mean ± standard deviation. Cell counting was performed in five randomly selected separate areas under ×20 magnification. *P < 0.05, compared with control; ^#^P < 0.05, compared with pantoprazole at 160 μg/ml (n = 5). **c** Wound closure assay. Upper panel: representative images of assay. Black bar indicates 500 μm. Lower panel: Comparison of vertical wound distance. Data are presented as mean ± standard deviation. All experiments were performed in triplicates. *P < 0.05, compared with control; ^#^P < 0.05, compared with pantoprazole at 160 μg/ml (n = 3). **d** 3-D culture spheroid cell invasion assay. Upper panel: representative images of 3-D culture spheroid invasion assay. Black bar indicates 200 μm. Lower panel: comparison of area of spheroids. Data are presented as mean ± standard deviation. All experiments were performed in triplicates. *P < 0.05, compared with control; ^#^P < 0.05, compared with pantoprazole at 40 μg/ml (n = 3). PPZ, pantoprazole; PV, pervanadate

**Fig. 5:**
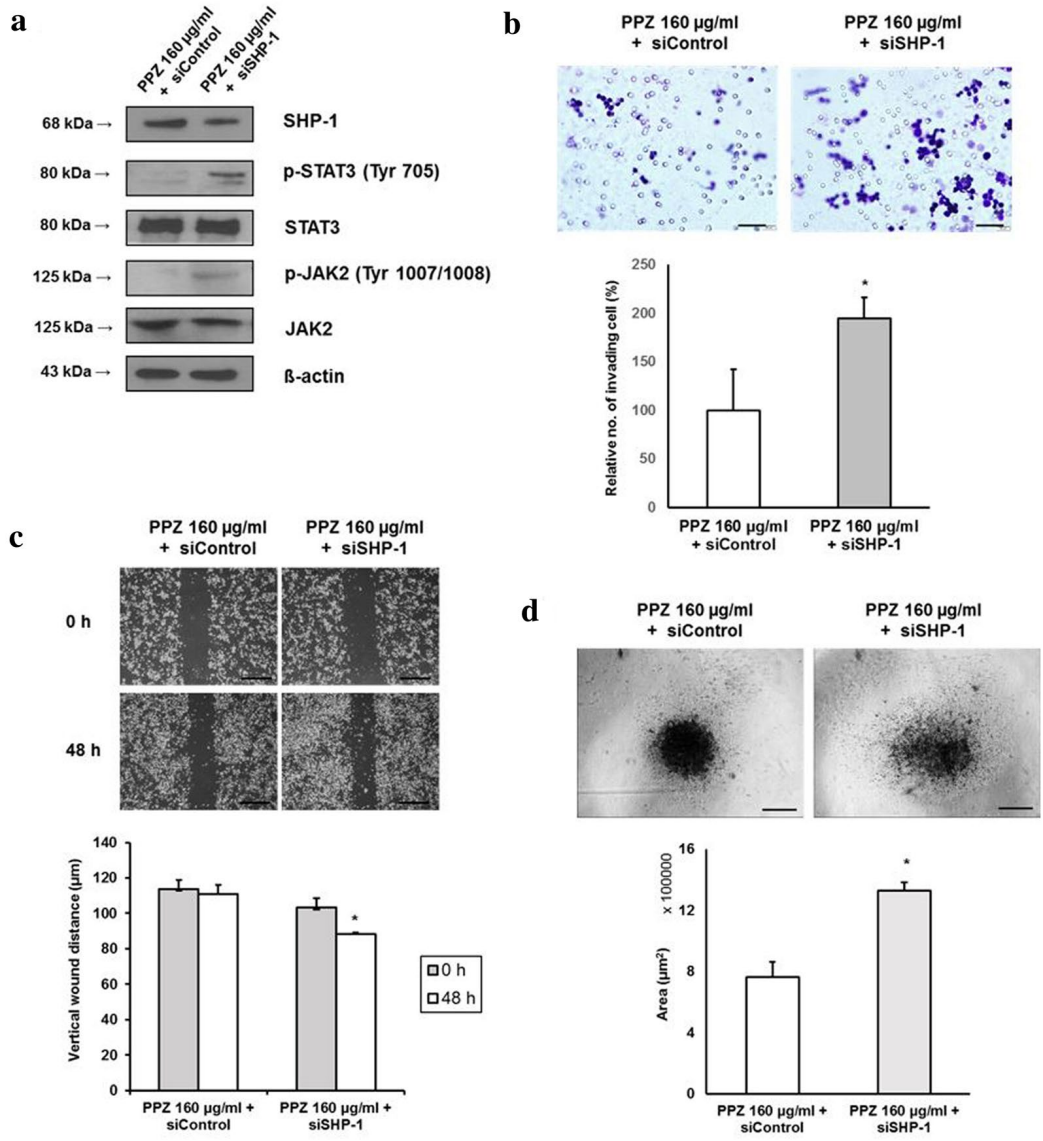
Inhibitory effects of pantoprazole on STAT3 activity, cellular migration, and invasion are attenuated by silencing of SHP-1. **a** Western blot after transfection and 48 h of treatment with pantoprazole at 160 μg/ml. **b** Matrigel invasion assay. Upper panel: representative images assay. Black bar indicates 50 μm. Lower panel: Comparison of invading cells. Data are presented as mean ± standard deviation. Cell counting was performed in five randomly selected separate areas under ×20 magnification. *P < 0.05, compared with pantoprazole 160 μg/ml + siControl (n = 5). **c** Wound closure assay. Upper panel: representative images assay. Black bar indicates 200 μm. Lower panel: Comparison of vertical wound distance. Data are presented as mean ± standard deviation. All experiments were performed in triplicates. *P < 0.05, compared with pantoprazole 160 μg/ml + siControl (n = 3). **d** 3-D culture spheroid cell invasion assay. Upper panel: representative images of 3-D culture spheroid invasion assay. Black bar indicates 200 μm. Lower panel: comparison of area of spheroids. Data are presented as mean ± standard deviation. All experiments were performed in triplicates. *P < 0.05, compared with control; ^#^P < 0.05, compared with pantoprazole at 40 μg/ml (n = 3). PPZ, pantoprazole

To validate mediating action of SHP-1 in the inhibition of STAT3 and cellular migration or invasion in gastric cancer cells, pervanadate, a pharmacologic phosphatase inhibitor, was used in this study. Pervanadate has been used in previous studies to validate the dephosphorylating action of SHP-1 on STAT3-activated malignant cells [12, 13]. Treatment with pantoprazole at 160 µg/ml for 48 h upregulated SHP-1, downregulated p-JAK2/p-STAT3 expression and modulated EMT markers (downregulation of Snail1, vimentin and upregulated E-cadherin) in AGS cells. This modulating effects were attenuated by pretreatment with 50 µM pervanadate (Fig. 4a). Matrigel invasion assay and wound closure assay also showed that pantoprazole at 160 µg/ml significantly decreased the number of invading cells and increased vertical wound distance. These inhibitory effects were also attenuated by pretreatment with pervanadate at 50 μM (Fig. 4b, c). The 3-D spheroid cell invasion assay showed that pantoprazole at 160 µg/ml reduced the spindle-like projection of AGS cells. This invasiveness was suppressed by pretreatment with 50 µM of pervanadate (Fig. 4d). Knockdown of SHP-1 by transfection of SHP-1 siRNA in AGS cells upregulated p-JAK2/p-STAT3 expression (Fig. 5a), attenuated anti-cellular migration and invasion effect by pantoprazole (Fig. 5b, c) and enhanced spindle-like cellular projection (Fig. 5d), which showed same effects with pervanadate. Taken together, our data suggest that SHP-1 might have a mediating role in the inhibitory effect of pantoprazole on STAT3 activity and migration or invasion of gastric cancer cells.

### SHP-1 mediates anti-tumor effect of pantoprazole

**Fig. 6:**
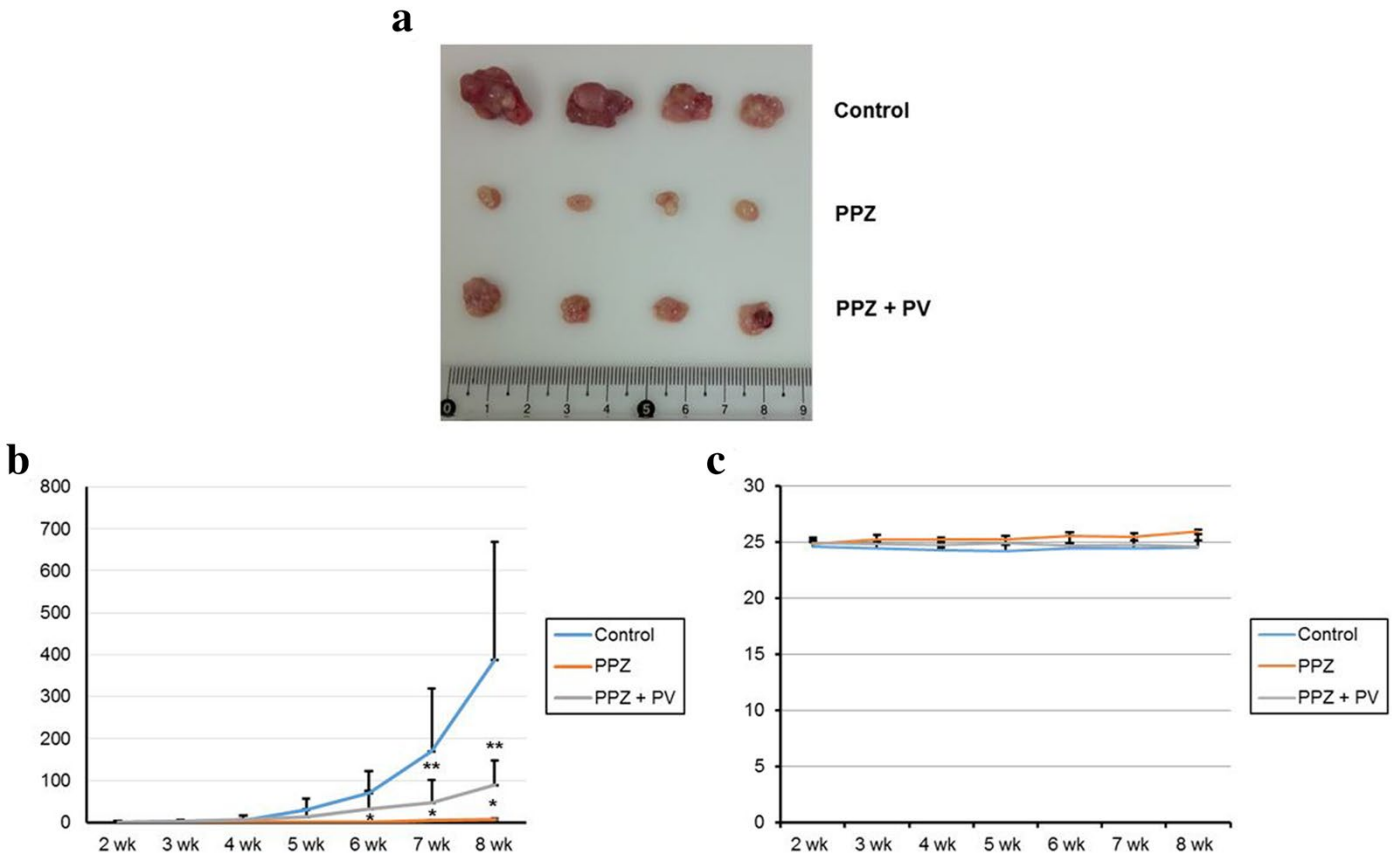
Antitumor effects of pantoprazole on xenograft tumor model. **a** Gross tumor extracted from nude mouse after 8 weeks of intraperitoneal administration of pantoprazole with or without pervanadate. **b** Mean tumor volume. Data are presented as mean ± standard deviation. *P < 0.05, compared with control group; **P < 0.05, compared with pantoprazole group (n = 4). **c** Changes of mean body weight. Data are presented as mean ± standard deviation. PPZ, pantoprazole; PV, pervanadate

**Fig. 7:**
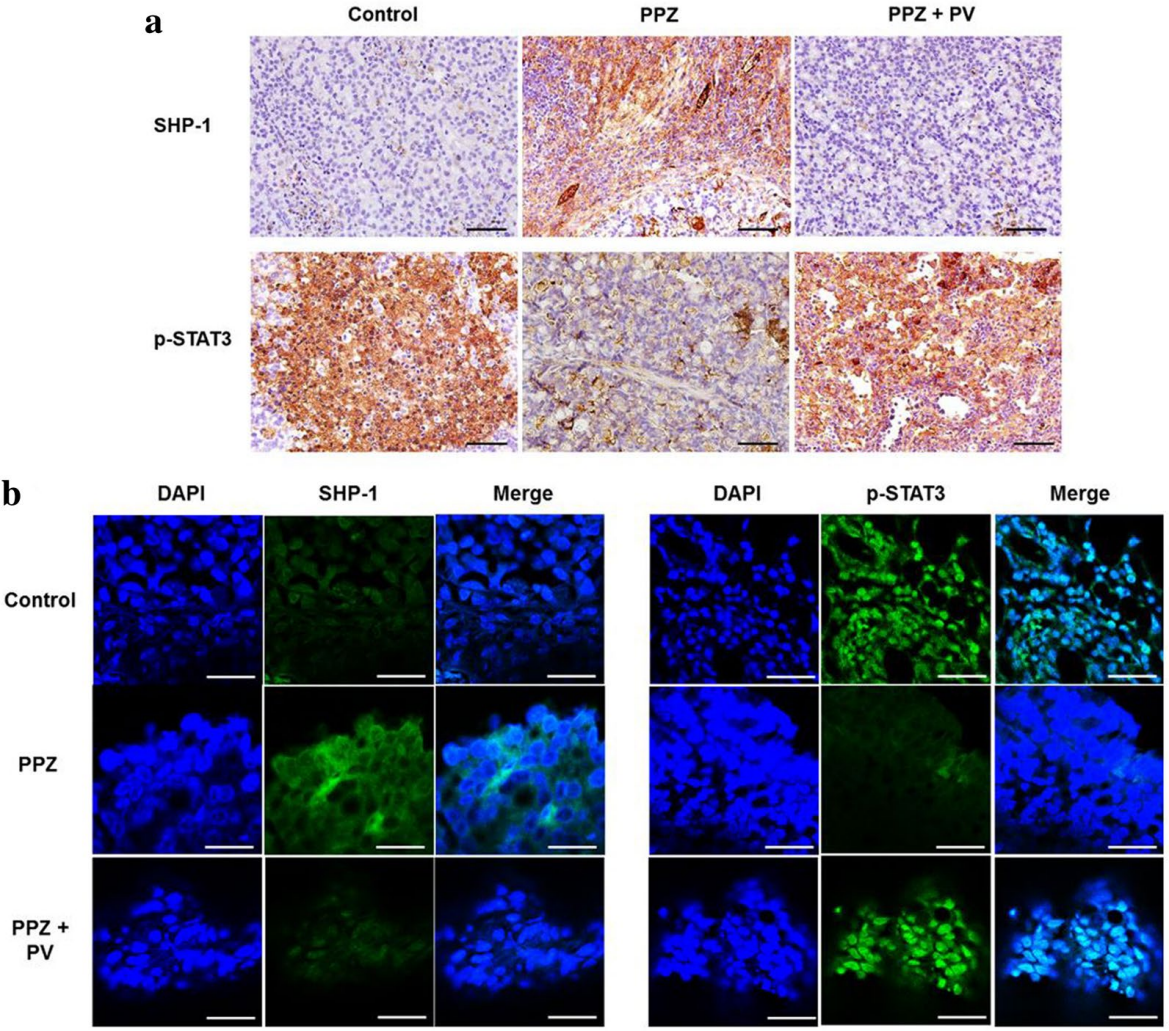
Pantoprazole upregulates SHP-1 expression and downregulates p-STAT3 expression in xenograft tumor. **a** Immunohistochemistry. SHP-1 and p-STAT3 protein expressions are stained dark brown. Black bar indicates 50 μm. **b** Immunofluorescence. Tumors are stained with SHP-1 (green), p-STAT3 (green), and 4′,6-diamidino-2-phenylindole (DAPI) (blue). White bar indicates 20 μm. PPZ, pantoprazole; PV, pervanadate

The xenograft tumor model showed that pantoprazole significantly reduced tumor volume compared to control. However, co-administration of pervanadate with pantoprazole resulted in significant increase in tumor volume compared to the control (Fig. 6a, b). There was no significant difference in body weight among the three groups (Fig. 6c). By immunohistochemistry stain, we observed intraperitoneal injection of pantoprazole enhanced SHP-1 expression and attenuated p-STAT3 expression compared to the control, however, concomitant administration of pervanadate attenuated this effect (Fig. 7a). Immunofluorescence staining also showed that pantoprazole upregulated cytosolic staining of SHP-1 and downregulated nuclear staining of p-STAT3. Such effects were reversed by co-administration of pervanadate with pantoprazole (Fig. 7b). Our results suggest that the anti-tumorigenic effect of pantoprazole might be mediated by the modulation of SHP-1/p-STAT3 axis in gastric cancer.

## Discussion

In this study, we observed that treatment with pantoprazole induced the expression of SHP-1 and downregulated the level of p-JAK2/p-STAT3. It also modulated EMT markers (upregulation of E-cadherin and downregulation of Snail1 and vimentin). Pantoprazole also showed significant anti-invasion and anti-migration effects. Modulation effects on SHP-1/p-JAK2/p-STAT3 and EMT markers by pantoprazole were attenuated by phosphatase inhibitor or silencing of SHP-1. In xenograft tumor model, pantoprazole significantly reduced tumor size and volume. Such effect was attenuated by concomitant injection of phosphatase inhibitor. Taken together, our results suggest that pantoprazole downregulates JAK2/STAT3 signaling through inducing SHP-1 expression to modulate snail/E-cadherin expression and inhibit cellular migration or invasion in gastric cancer cells.

Inhibiting STAT3 activity is an important issue for treating gastric cancer. Various types of substances or drugs that target STAT3 have been developed and studied in basic research and clinical trials for many years. However, most STAT3 inhibitors have been evaluated in experimental studies. Direct inhibition of STAT3 includes targeting Src homology 2 (SH2) domain [14], DNA binding domain, or N-terminal domain of STAT3, oligonucleotide-based inhibition of STAT3 such as siRNA, or decoy oligonucleotide technology [15]. Indirect inhibition of STAT3 includes targeting upstream intracellular kinases such as JAK2 or Src kinase [16, 17]. However, limitations of these STAT3-inhibition strategies is that most of these substances have been only evaluated in pre-clinical studies. Few agents have been evaluated in phase II or III clinical trials. Clinical outcomes including sufficient number of gastric cancer patients are especially lacking. Technical difficulties are also encountered in the attempt to develop more suitable and effective agents that can directly target STAT3. Thus, new strategy for inhibiting STAT3 activity is needed.

Inhibitory effects of SHP-1 on JAK2/STAT3 signaling pathway have been previously reported in hematopoietic malignancies. Indeed, SHP-1 expression is very abundant in hematopoietic cells. It is one of the most important regulators of cellular signaling in lymphocytes and myeloid cell lineage, including macrophages, neutrophils, monocytes, and mast cells [18]. Most previous studies concerning the impact of SHP-1 have focused on the tumor suppressive function of SHP-1 or aberrantly reduced expression of SHP-1 by CpG island promoter hypermethylation in lymphoma or leukemia [19]. However, few studies have demonstrated aberrant mRNA expression of SHP-1 in epithelial cancer cell lines except estrogen receptor-negative breast cancer, prostate cancer, or pancreas cancer cell lines [20, 21]. In epithelial cells of gastrointestinal tract, a previous study has shown that CpG promoter hypermethylation of SHP-1 is frequently observed in colorectal cancer cells while demethylating agents such as DNA methyltransferase inhibitor and 5-Aza-2′-deoxycytidine can induce SHP-1 expression to downregulate JAK2/STAT3 signaling [22]. Concerning gastric cancer, methylation rate of SHP-1 promoter has been reported to be 25% or 40–70% in gastric carcinoma tissues [23]. However, underlying mechanism of aberrant SHP-1 expression in gastric cancer has not been fully elucidated yet. Recently, Sun et al. have shown that the transmembrane protein with epidermal growth factor and two follistatin motifs 2 (TMEFF2) is a key co-factor of SHP-1. The both cooperatively work to inhibit phosphorylation of STAT3 in gastric cancer cells. IHC staining showed overlapped expression of TMEFF2 and SHP-1 in the cytosol. Downregulation of SHP-1 markedly blocked TMEFF2-induced decrease in cell proliferation and increase in cell apoptosis. These authors concluded that TMEFF2 could act as a tumor suppressor in gastric cancer through direct interaction with SHP-1 via its intercellular domain [24]. A recent pivotal study has demonstrated that SHP-1 interacts and dephosphorylates Glu-Pro-Ile-Tyr-Ala (EPIYA) motifs of CagA of *H. pylori*. Infection of gastric epithelial cells with Epstein-Barr virus (EBV) can inactivate SHP-1 via promoter hypermethylation which enhances *H. pylori* CagA activity and the development of EBV-associated gastric cancer [25].

Baseline expression of SHP-1 is very weak in most gastric cancer tissues or cell lines. Thus, SHP-1-induction strategy appears to be reasonable to effectively inhibit constitutive expression of STAT3. Recent studies have reported that several natural compounds show significant STAT3 inhibition and anti-invasive or metastatic effects by mediating SHP-1 activity [26, 27]. Several other drugs or compounds can also effectively dephosphorylate JAK2/STAT3 by inducing SHP-1 expression in gastric cancer, including multiple kinase and other natural compounds. Pantoprazole is one of most commonly used PPI. It also showed unexpected effects such as anti-proliferation or enhancing chemosensitivity in vitro by inhibiting STAT3 [28]. However, the underlying mechanism involved in STAT3 inhibition by pantoprazole has not been investigated yet. Our data suggest that SHP-1 may play a pivotal role in downregulating STAT3 induced by pantoprazole in gastric cancer cells. Thus, pantoprazole, a commonly used drug, might have a potential as an effective SHP-1 inducer to inhibit STAT3 activity and overcome chemoresistance. Indeed, a recent phase II study has shown that intermittent high dose PPI in conjunction with systemic chemotherapy can enhance the antitumor effect in metastatic breast cancer patients [29]. However, our study has several limitations. First, a specific mechanism of pantoprazole or other PPIs to activate SHP-1 is not elucidated. A recent study showed that luteolin, a plant flavonoid, disrupted the binding of heat-shock protein-90 (HSP-90) to STAT3 and induced the interaction between SHP-1 and STAT3 and dephosphorylation of STAT3 [26]. Several previous studies showed that the expression of HSPs in gastric cancer cells and rat gastric mucosa were regulated by PPI treatment [30, 31], and HSPs might be a potential molecular link between pantoprazole and induction of SHP-1, which needs to be further investigated. Second, pantoprazole is also reported to suppress other pathways including Wnt/β-catenin signaling that is involved in EMT process in gastric cancer cells [32, 33]. The inhibitory effects of pantoprazole on cellular invasion or migration and modulation of EMT markers may not be solely dependent on its action on SHP-1/STAT3 signaling axis. Future researches may aim the following: (1) effect of enhancing SHP-1 on angiogenesis or cancer-associated fibroblasts in gastric cancer since constitutive STAT3 activation is crucial for creating the tumorigenic microenvironment such as angiogenesis and fibrogenesis [34]. (2) effect of SHP-1 on downregulation of phosphoinositide 3-kinase (PI3K)/Akt pathway in gastric cancer since SHP-1 might have potential to modulate other signaling pathways such as nuclear factor kappa-light-chain-enhancer of activated B cells (NF-κB) and PI3K/Akt pathways via dephosphorylation of receptor tyrosine kinases [5, 35].

## Conclusion

In summary, SHP-1 as an effective phosphatase for inactivation of JAK2/STAT3 might be applied in gastric cancer and pantoprazole showed significant modulating effect on SHP-1/JAK2/STAT3 signaling axis in stomach cancer. More effective enhancer of SHP-1 and its underlying mechanisms in gastric cancer need to be investigated in the future.

## Abbreviations

JAK2: janus kinase 2
STAT3: signal transducer and activator of transcription 3
*H. pylori*: *Helicobacter pylori*
SHP-1: SH2-containing protein tyrosine phosphatase 1
PPI: proton pump inhibitor
RNA: ribonucleic acid
IHC: immunohistochemistry
mRNA: messenger RNA
EBV: Epstein–Barr virus
PI3K: phosphoinositide 3-kinase
NF-κB: nuclear factor kappa-light-chain-enhancer of activated B cells
